# Pyogenic Flexor Tenosynovitis in an Infant

**DOI:** 10.5811/cpcem.2016.12.31521

**Published:** 2017-03-13

**Authors:** James I. Gragg, Ryder Olsen, S. Briant Stringham

**Affiliations:** Carl R. Darnall Army Medical Center, Department of Emergency Medicine, Fort Hood, Texas

## Abstract

Pyogenic flexor tenosynovitis is a rare, though well known infectious process of the flexor tendon sheath of the hand. This condition is generally diagnosed in adults by the observance of the four Kanavel signs. Application of the Kanavel signs to diagnosis in the pediatric population, however, is of unknown utility. We present the case of a 13-month-old male with pyogenic flexor tenosynovitis who presented with all four of the Kanavel signs.

## INTRODUCTION

Pyogenic flexor tenosynovitis (PFT) is an acute or subacute infectious process of the flexor tendon sheath of the digits. Dr. Kanavel first described the process in 1912 in his textbook on surgical infections of the hand. At the time he outlined three cardinal signs and symptoms of the disease: excessive tenderness over the course of the sheath; symmetric enlargement of the whole finger; excruciating pain on extending the finger.^1^ Later, he described the finger being held slightly flexed.^1^ Together, these four signs and symptoms are now known as the Kanavel signs and should lead the clinician to suspect pyogenic infection of the tendon sheath.

There are three main mechanisms of infection, the most common being direct inoculation from trauma, laceration, or bite. Less frequently, contiguous spread from a local infection such as a felon or paronychia, or hematogenous spread may occur. Precipitous spread of the infection occurs due to the avascular nature of the synovial sheath. The sheath receives all blood supply from surrounding tissue; as such, the infection is relatively protected from the immune response.^2^ Rapid assessment and diagnosis is imperative to prevent both short-term and long-term sequelae: stiffness, loss of motion across the inter-phalangeal joints, deformity with soft-tissue loss, osteomyelitis, or spread of infection with resultant amputation.^3^

Unfortunately, despite the use of the Kanavel signs over the past century, there are no studies to validate the sensitivity or specificity of these signs, nor their ability to positively diagnose PFT. The disease has a low incidence, and lack of studies is likely related to limited patient presentations with the resultant non-feasibility of prospective trials. In one retrospective review of 75 cases of PFT, fusiform swelling was found in 97%, pain with passive extension in 72%, flexed position in 69% and tenderness along the tendon sheath in 64% of patients.^4^ This report looked only at adult patients, however, and there are minimal reports of tenosynovitis in the pediatric population. As such, the reliability of the Kanavel signs for diagnosis in pediatric patients is of unknown certainty.

## CASE REPORT

We present a rare case of PFT in a 13-month-old male. The patient presented during a morning shift to the emergency department (ED) with the chief complaint of finger swelling. His father reported a swollen and erythematous right index finger that started roughly four hours prior to arrival in the ED. The night before he had gone to bed uneventfully, with a normal appearing hand. He awoke tearful the next morning and was brought to the ED for evaluation. The father denied any known injuries or periods of unobserved play.

**Image 1:**
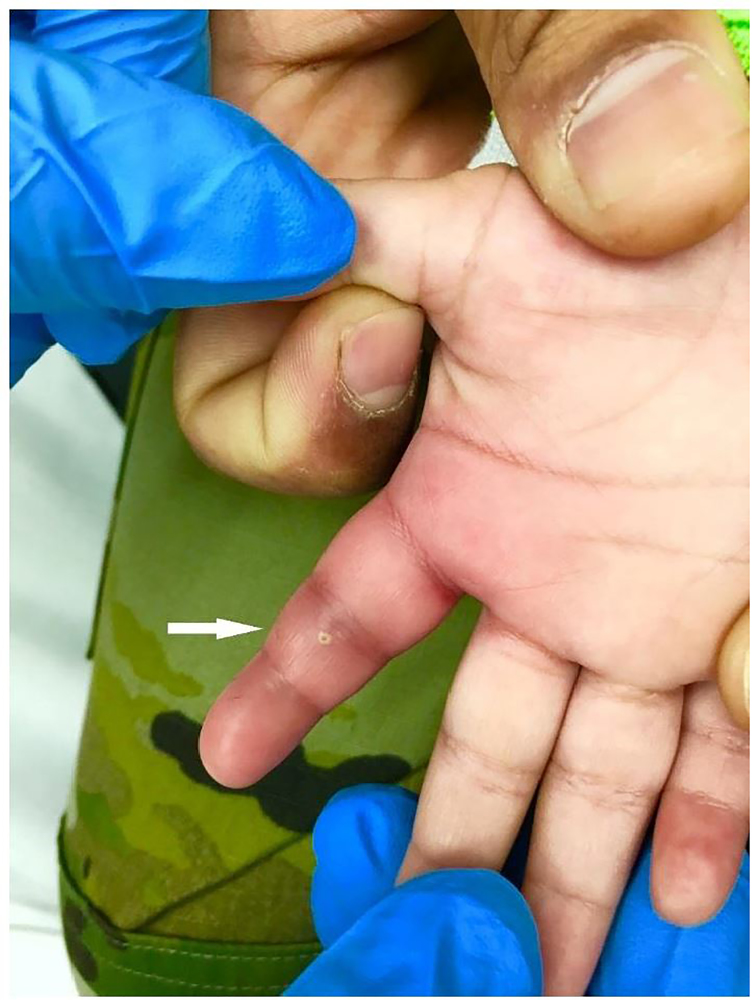
Right index finger with fusiform swelling and erythema. Note the pustule over the proximal inter-phalangeal joint (arrow).

**Image 2:**
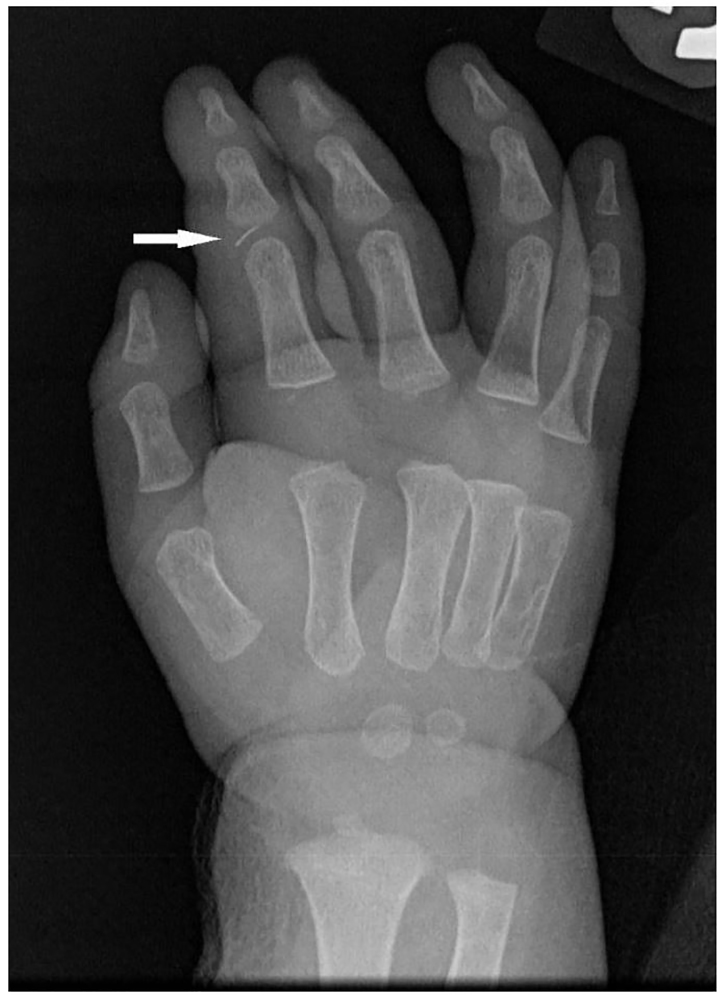
Radiograph of right hand: Note the foreign body within the index finger proximal inter-phalangeal joint (arrow).

On physical exam, all four Kanavel signs were present. The finger was held in flexion and circumferentially swollen from fingertip to metacarpal-phalangeal joint (Image 1). When the flexor surface of the finger was palpated or passively extended, the child immediately cried and appeared distressed. A pustule was also noted on the palmar surface over the proximal interphalangeal (PIP) joint. He was afebrile with normal vital signs, appropriate interactions and non-distressed when left to his own accord. Laboratory studies demonstrated a total leukocyte count of 13.3 x10^9^ cells/L, an erythrocyte sedimentation rate of 25 mm/hr, and a C-reactive protein level of <0.5 mg/L. On radiograph, a metallic foreign body was identified near the PIP joint (Image 2).

With concern for PFT, orthopedics consultation was obtained and the patient was started on intravenous clindamycin. He was taken to the operating room (OR) emergently for washout and foreign body removal. Pus was noted to be draining from the subcutaneous tissue, but the flexor tendon sheath was not felt to be involved at that time. Unfortunately, the patient had clinical worsening and was taken back to the OR two days later. During the second surgery purulent material was noted within the flexor tendon sheath tracking proximally to the distal palmar crease. Another very small radiolucent foreign body was also discovered and removed. Group A *Streptococcus* was isolated from his wound culture and after an uneventful 24 hours of observation, he was discharged home on a course of oral antibiotics. A single blood culture was negative after five days of growth. On post-operative day 10, a clinic follow-up visit demonstrated well healing surgical wounds and absence of any of the Kanavel signs.

## DISCUSSION

PFT is a serious medical condition requiring emergency surgery to halt progression of infection and limit potential sequelae. While a rare entity in the general population, there are even less data on pediatric tenosynovitis. A case series of only three cases has suggested that the infection is typically preceded by a penetrating injury.^5^ While there was no known injury in our case, the presence of a foreign body and pustule highly suggests a penetrating injury. Additional unique risk factors in the pediatric population include thumb sucking and fingernail biting.^6^ The local trauma induced from these habits weakens the integumentary barrier and can facilitate infection. Not surprisingly, *Staphylococcus* and *Streptococcus* are the most commonly isolated organisms in the adult population, a trend also seen in pediatric hand infections.^6^ Antibiotics should, therefore, be targeted against natural skin flora while awaiting surgical infection control.

A high index of suspicion is needed to make the diagnosis of PFT. Definitive diagnosis is confirmed by wound culture, although the clinical presentation suggests the need for surgery. As mentioned, the Kanavel signs have not been prospectively validated in children. The Luria and Haze case series had two cases presenting with all four Kanavel signs and another case presenting with only two.^5^ Interestingly, the last case presented with the fingers held in extension rather than the expected flexion.^5^ Our patient did have all four signs present on admission.

Additionally, patients will typically be afebrile and radiographs are usually normal, making the diagnosis more difficult. While the clinical presentation may be sufficient to proceed directly with surgery, other imaging modalities may help in the evaluation. Magnetic resonance imaging, if available, may show fluid collection in the tendon sheath.^7^ Point-of-care ultrasound is another option for the evaluation of indeterminate cases. A stand-off pad or water bath is best used to identify hypoechoic fluid within the tendon sheath; comparison to the normal contralateral digit will aid the interpretation.^8^ Ultimately the decision to proceed with surgery is made by the consulting service; however, the emergency physician should not hesitate to begin intravenous antibiotics.

## CONCLUSION

PFT is a rare infection that can be easily mistaken for other infectious states such as cellulitis or septic arthritis. This report demonstrates that the Kanavel signs may help the clinician with early identification of PFT in the pediatric population and facilitate prompt treatment.

## References

[1] Kanavel AB (1912). Infections of the Hand.

[2] Draeger RW, Bynum DK (2012). Flexor tendon sheath infections of the hand. J Am Acad Orthop Surg.

[3] Kennedy CD, Huang JI, Hanel DP (2016). In Brief: Kanavel’s Signs and Pyogenic Flexor Tenosynovitis. Clin Orthop Relat Res.

[4] Pang HN, Teoh LC, Yam AK (2007). Factors affecting the prognosis of pyogenic flexor tenosynovitis. J Bone Joint Surg Am.

[5] Luria S, Haze A (2011). Pyogenic flexor tenosynovitis in children. Pediatr Emerg Care.

[6] Harness N, Blazar PE (2005). Causative microorganisms in surgically treated pediatric hand infections. J Hand Surg Am.

[7] Ceroni D, Merlini L, Salvo D (2013). Pyogenic flexor tenosynovitis of the finger due to Kingella kingae. Pediatr Infect Dis J.

[8] Cohen SG, Beck SC (2015). Point-of-Care Ultrasound in the Evaluation of Pyogenic Flexor Tenosynovitis. Pediatr Emerg Care.

